# Vision‐related quality of life and visual ability in patients with autosomal dominant optic atrophy

**DOI:** 10.1111/aos.15102

**Published:** 2022-02-10

**Authors:** Christina Eckmann‐Hansen, Toke Bek, Birgit Sander, Michael Larsen

**Affiliations:** ^1^ Department of Ophthalmology Rigshospitalet Glostrup Denmark; ^2^ Faculty of Health and Medical Sciences University of Copenhagen Copenhagen Denmark; ^3^ Department of Ophthalmology Aarhus University Hospital Aarhus Denmark

**Keywords:** autosomal dominant optic atrophy, CVAQC, NEI‐VFQ, quality of life, visual function

## Abstract

**Purpose:**

The purpose of the study was to evaluate vision‐related quality of life and visual ability in patients with *OPA1* autosomal dominant optic atrophy (ADOA).

**Methods:**

This cross‐sectional, observational study included 145 participants with a mutation in the *OPA1* gene associated with ADOA, 63 mutation‐free first‐degree relatives and 92 healthy subjects unrelated to the families. Participants underwent a clinical eye examination, and adult participants completed the National Eye Institute Visual Function Questionnaire (NEI‐VFQ‐39), while children completed the Cardiff Visual Ability Questionnaire for Children (CVAQC).

**Results:**

In adults with ADOA, both mean visual acuity (VA) and mean contrast sensitivity (CS) were significantly inferior to both first‐degree relatives and unrelated controls (p < 0.001). In children with ADOA, mean VA was significantly lower compared with first‐degree relatives (p = 0.0052), whereas CS was not (0.127). Adults with ADOA scored lower than both comparator groups on composite score (p < 0.001), general health subscale (p = 0.0075) and all vision‐related subscales (p < 0.001) except the ocular pain subscale (p = 0.2). In children with ADOA, the median CVAQC logit score was significantly lower compared with first‐degree relatives (p = 0.037). The science lessons subscale was significantly lower for children with ADOA compared with first‐degree relatives (p = 0.046), as well as the language lessons subscale (p = 0.038). For adults, composite score and subscale scores were significantly associated with both VA, CS and fixation status.

**Conclusion:**

*OPA1* mutation is associated with lower quality of life and visual ability in patients with ADOA compared with both first‐degree relatives and unrelated controls. VA, CS and fixation status affect quality of life in patients with ADOA.

## Introduction

Autosomal dominant optic atrophy (ADOA) is the most common inherited optic neuropathy in Denmark with a prevalence of 1:10 000 (Kjer *et al*. 1996). At present, there is no documented cure for the disease, which is caused by a mutation in the *OPA1* gene and causes bilateral visual impairment. Patients with ADOA experience decreased vision due to cecocentral scotomas that affect both distance and near vision. The variability of visual function in ADOA is large, both among and within families, ranging from normal visual function to severe visual impairment in patients with substantial loss of inner retinal cells (Amati‐Bonneau *et al*. 2009, Lenaers *et al*. 2012, Rönnbäck *et al*. 2015). It is known that other hereditary ophthalmic diseases that affect foveal vision, such as Stargardt disease, cone dystrophy and Leber's hereditary optic neuropathy are associated with reduced quality of life (Kirkman *et al*. 2009, Roh *et al*. 2018, Sahli *et al*. 2020).

The aim of this study was to evaluate vision‐related quality of life in patients with ADOA with reference to healthy first‐degree relatives and healthy individuals unrelated to the families affected by ADOA. Quality of life was assessed in adults using the National Eye Institute Visual Function Questionnaire (NEI‐VFQ‐39) and visual ability in children using the Cardiff Visual Ability Questionnaire for Children (CVAQC). The results provide quantitative and qualitative information about visual challenges experienced by patients with ADOA.

## Materials and Methods

This descriptive, cross‐sectional study included 331 participants divided into three groups: a group with ADOA (*n* = 158) a group of healthy first‐degree relatives (*n* = 81) and a group of healthy control subjects unrelated to the families affected by ADOA (*n* = 92). Patients and relatives from 48 different families known with ADOA were included. The first‐degree relatives can reasonably be assumed to have had a higher degree of shared environment with participants with *OPA1* mutation than what is true for the unrelated controls. Study procedures were carried out at Rigshospitalet in Copenhagen (244 participants) and at Aarhus University Hospital (87 participants). The first two groups were open to participants of any age, while the latter admitted only participants aged 18 years or older. All participants were examined by the same investigator (CEH). Patients and first‐degree relatives were invited through a national register. Unrelated healthy control participants were recruited through a dedicated website (www.forsøgsperson.dk). Written informed consent was obtained from adults and adolescents aged 15 years or older and, for children under the age of 15 years, from their parents or legal guardians. The study was approved by the local medical ethics committee, the Danish Data Protection Agency and the Danish Patient Safety Authority and adhered to the tenets of the Declaration of Helsinki. Inclusion in the *OPA1* ADOA group required laboratory evidence of an *OPA1* mutation. Defects in *OPA1* included c.2826_2836delinsGGATGCTCCA (*n* = 56), c.983A > G (*n* = 28), c.2708_2711delTTAG (*n* = 20), c.2728_2730delGTT (*n* = 7), c.2614‐9A > G (*n* = 6), c.2496 + 4_2496 + 5delinsGTAAC (*n* = 5), c.(2818 + 1_2819–1)_(2883+ 1_*6‐1)del (*n* = 4), c.(2496 + 1_2497–1)_(2707 + 1_2708‐1)del (*n* = 3), c.(32 +1_33–1)_(678 + 1_679‐1)del (*n* = 3), c.1516 + 5G > A (*n* = 3), c.[1096C > T];[=] (*n* = 2), c.2707 + 1G > C (*n* = 2), c.[267G > A];[=] (*n* = 1), c.[870 + 2 T > A];[=] (*n* = 1), c.1304_1305delGT (*n* = 1), c.1687C > T (*n* = 1), c.2496G > C (*n* = 1) and c.2713C > T (*n* = 1). Inclusion in the first‐degree relative group was parents, children and siblings of the former, in whom a normal *OPA1* genotype was found. A total of 145 patients and 63 first‐degree relatives were genetically verified.

All participants underwent a clinical examination, which included thorough refraction and best‐corrected visual acuity testing according to the Early Treatment Diabetic Retinopathy Study (ETDRS) protocol (Ferris *et al*. 1982). Additional tests included contrast vision (Pelli Robson contrast sensitivity with the exact count of read letters being included, not the total triplets of letters), biometry measurement (IOL Master 500, software version 7.1.2.042 or IOL master 700, software version 1.50, both Carl Zeiss Meditec, La Jolla, CA, USA), fundus photography (TRC‐50DX, IMAGEnet i‐base version 3.23.0, Topcon, Tokyo, Japan), spectral‐domain optical coherence tomography (HRA + OCT Spectralis OCT2, HRA2 software version 6.12.3.0 or HRA + OCT Spectralis OCT1, HRA2 version 6.9.4.0, software version 6.9a, both Heidelberg Engineering, Heidelberg, Germany), microperimetry (MAIA, Centervue, Padova, Italy, software version 2.5.1) and adaptive optics fundus photography (rtx1e, AO Image version 3.3 or rtx1, AO Image version 3.3, both Imagine Eyes, Orsay, France). [Correction added on 04 April 2022, after first online publication: The sentence 'The Pelli Robson contrast sensitivity was expanded in this current version.]

Fixation assessment was based on the standard protocol of the MAIA with a grid covering 10 degrees of the preferred fixation of the patients with stimuli at the centre, and at one, three and five degrees from the centre. Location of fixation was assessed by CEH and defined as a binary variable (central or eccentric) defined as central with majority of fixation points and the bivariate contour ellipse area inside the central five degrees of the central fovea in both eyes, and as eccentric with majority of fixation points outside in one or both eyes. Fixation stability was defined as a binary variable (stable or unstable) defined as the category ‘stable’ as stable fixation in both eyes and the two categories ‘relatively unstable’ and ‘unstable’ as unstabel fixation in one or both eyes. Area of preferred fixation was defined as the bivariate contour ellipse area that encompasses 95% of the fixation points within standard deviation horizontal and vertical eye positions during the attempt of fixation (BCEA95). In total, 546 eyes of adults and 46 eyes of children were included in the analysis of fixation. Three eyes in adults and three eyes in children were not examined with the MAIA due to impossible fixation caused by severe micro‐nystagmus, and one eye in each group was excluded due to at least three false‐positive responses.

Vision‐related quality of life was assessed using the validated Danish version of the National Eye Institute Visual Function Questionnaire (NEI‐VFQ‐39) (Sørensen *et al*. 2011). The questionnaire was self‐administered unless the participant was unable to read, in which case it was administered by the investigator. The Danish version contains a supplemental question about computer usage, but this question was not included in the final analysis in this study. Scores were calculated using the scoring manual, and all 39 questions and subgroups were included and analysed. Composite score was calculated as a mean of all the subscales. Completely missing questionnaires from three patients, one first‐degree relative and three healthy control subjects were omitted in the analysis, which resulted in 124 participants with ADOA, 55 first‐degree relatives and 89 unrelated controls being included in the statistical analysis of the NEI‐VFQ‐39.

For the children, visual ability was assessed using a Danish version of Cardiff Visual Ability Questionnaire for Children (CVAQC), which has not yet been validated (Hansen *et al*. 2020). The questionnaire was self‐administered when participants were fluent readers or administered by a parent questioning the child. A geography class question was omitted from the Danish version as it failed to make sense after translation and was scored as a missing value. A question about tablet usage that has been added to the Danish version of the questionnaire was omitted from the analysis because it has no representation in the original test's scoring algorithm (Khadka *et al*. 2010). The median score on a logit scale was calculated using the scoring sheet. A ratio was calculated using the method described by Hansen et al. (2020) In the present study, an individual ratio was also calculated for each item for each participant by dividing the actual raw score with 4, thus, the scores range from 1 to 4.

For statistical analysis, the best‐corrected visual acuity of only the better seeing eye was used in participants with amblyopia or other unilateral ocular abnormality. Descriptive analyses of visual acuity and contrast sensitivity were otherwise made using the mean of the two eyes. Statistical analysis of NEI‐VFQ‐39 and CVAQC data was made using a mixed model, correcting for family as a random effect. All models were corrected for age and sex. Results were calculated with the statistical software RStudio version 1.2.5001. Mixed models were applied using the nlme package (RStudio Team 2019, version 3.1–150). Radar plots were created using the ggRadar package (Bion 2020, version 0.2). Unless stated otherwise, p‐values are reported for the comparison of participant groups (3 for adults, 2 for children). Pairwise comparisons were made whenever relevant using the same model. p‐Values below 0.05 were considered statistically significant.

## Results

In adults, mean age was higher in participants with *OPA1* mutation, compared with both mutation‐free first‐degree relatives and unrelated control subjects. Sex was equally distributed in the groups. Adult participants with *OPA1* mutation had markedly lower visual acuity and contrast sensitivity than both mutation‐free first‐degree relatives and unrelated control subjects. The two control groups were equal on all parameters. See Table 1.

**Table 1:** Characteristics of adult participants. [Correction added on 12 April 2022, after first online publication: Table 1 was corrected in this version.]

	ADOA (*n* = 127)	First‐degree relatives (*n* = 56)	Unrelated control subjects (*n* = 92)	p	Intergroup comparison		
	Mean/N (range)	Mean/N (range)	Mean/N (range)		p ADOA vs first‐degree relatives	p ADOA vs unrelatedcontrols	p First‐degree relatives vs. unrelated controls
Age (years)	48.4 (19;86)	41.3 (18;84)	41.7 (19;80)	0.0027	0.0085	0.0048	0.9
Sex (male/female)	67/60*	21/35*	40/52*	0.1	N/A	N/A	N/A
Visual acuity (ETDRS letters)	56 (3;99)	89 (58;97)	90 (72;100)	<0.001	<0.001	<0.001	0.8
Contrast sensitivity (log CS)	1.21 (0.1;1.65)	1.56 (1.3;1.73)	1.58 (1.33;1.83)	<0.001	<0.001	<0.001	0.6
Fixation (central/eccentric)	206/45^†^	112/0^†^	183/0^†^	<0.001	N/A	N/A	N/A
Fixation stability (stable/unstable)	182/69^†^	111/1^†^	181/2^†^	<0.001	N/A	N/A	N/A
Fixation area BCEA95 (°^2^)	7.2 (0.2;60.3)	1.8 (0.2;8.4)	1.6 (0.2;31)	<0.001	<0.001	<0.001	0.9

^*^ Number of persons.

^†^ Number of eyes.

In children with *OPA1* and their mutation‐free first‐degree relatives, the distributions of age and sex were comparable, albeit in a smaller sample than for adults. Children with ADOA had a lower visual acuity than the first‐degree relatives but similar contrast sensitivity. The *OPA1*‐associated deficit of 21 letters in children was considerably smaller than that of 33 letters in the adults. A similar association with age on the severity of ADOA was seen for contrast sensitivity. See Table 2.

**Table 2:** Characteristics of participants younger than 18 years.

	ADOA (*n* = 18)		First‐degree relatives (*n* = 7)		p
	Mean/N	Range	Mean/N	Range	
Age (years)	12.8	7;17	10.4	7;13	0.15
Sex (male/female)	7/11*	N/A	3/4*	N/A	0.86
Visual acuity (ETDRS letters)	65	44;85	86	75;91	0.0052
Contrast sensitivity (log CS)	1.31	0.75;1.58	1.45	1.35;1.55	0.127
Fixation (central/eccentric)	31/14^†^	N/A	1/0^†^	N/A	0.5
Fixation stability (stable/unstable)	30/12^†^	N/A	2/2^†^	N/A	0.4
Fixation area BCEA95 (°^2^)	4.8	0.7;16.2	5.7	1.2;15.4	0.9

^*^ Number of persons.

^†^ Number of eyes.

**Fig. 1:**
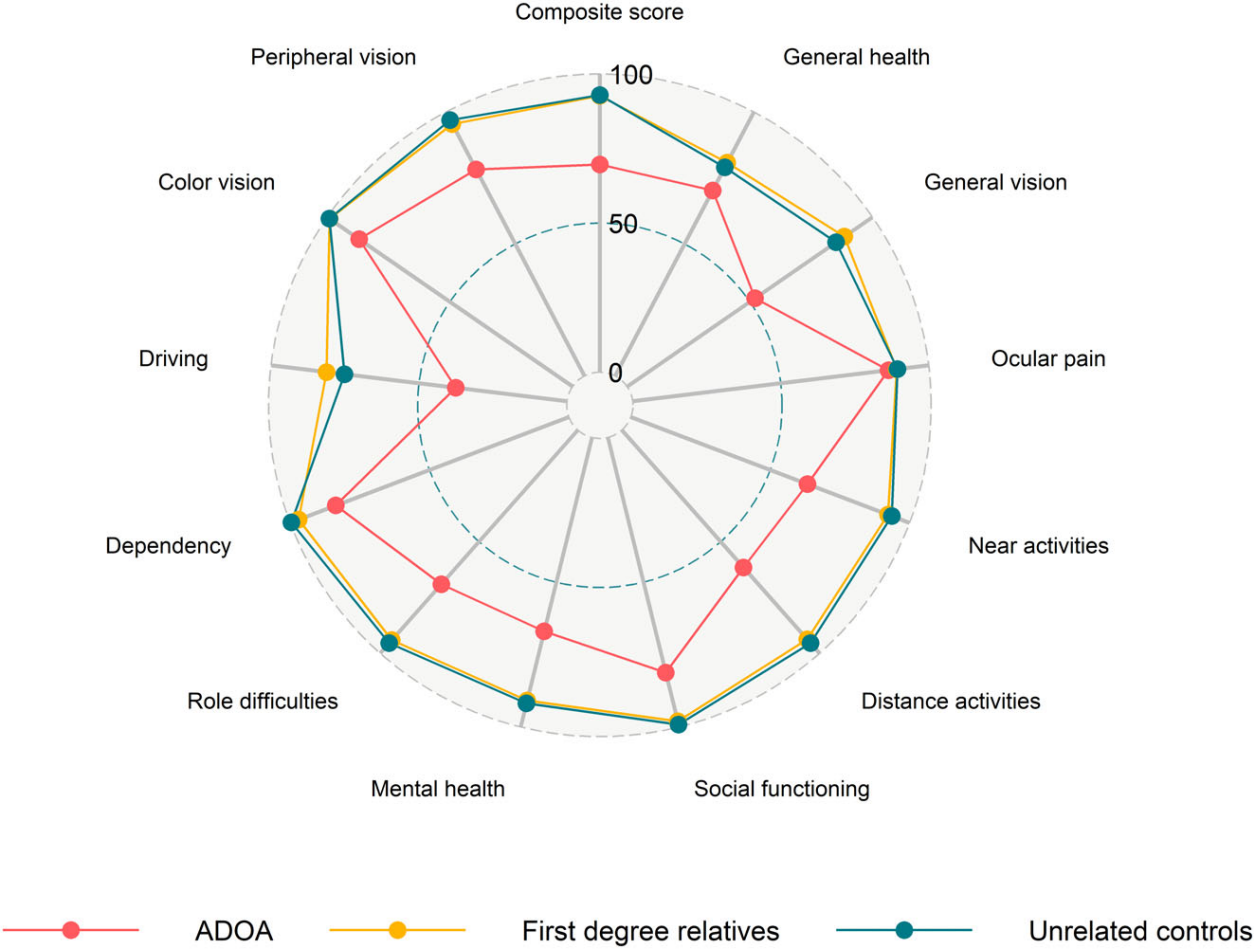
National Eye Institute Visual Function Questionnaire (NEI‐VFQ‐39) scores on vision‐related quality of life in adult participants with *OPA1* mutation (red), first‐degree relatives of the former (yellow) and unrelated healthy control participants (green) on arbitrary scales from 0 (worst) to 100 (best). [Colour figure can be viewed at wileyonlinelibrary.com]

Adults with *OPA1* mutation obtained a lower composite NEI‐VFQ‐39 compared with first‐degree relatives and unrelated controls (both p < 0.001). The ADOA‐associated deficit in daily visual function was broadly distributed across the 12 subcategories of the NEI‐VFQ‐39, except ocular pain, which was comparable for all three groups (Table 3, Fig. 1)). The most pronounced visual function deficits in ADOA were in the categories *driving* and *general vision*.

**Table 3:** Visual Function Questionnaire (NEI‐VFQ‐39) scores of adult participants. [Correction added on 12 April 2022, after first online publication: Table 3 was corrected in this version.]

	ADOA (*n* = 120)	First‐degree relatives (*n* = 55)	Unrelated controls (*n* = 89)	Overall comparison p	Intergroup comparison		
	Mean (range), n/a	Mean (range), n/a	Mean (range), n/a		p ADOA vs first‐degree relatives	p ADOA vs unrelatedcontrols	p First‐degree relatives vs. unrelated controls
Composite score	69.7 (10–100), 3	92.7 (54–100), 1	92.9 (68–100), 3	<0.001	<0.001	<0.001	0.9
General health	70.3 (13–100), 3	80.9 (33–100), 1	79 (38–100), 2	0.0075	0.0040	0.0113	0.5
General vision	52.1 (10–100), 3	88.6 (45–100), 1	85.2 (50–100), 2	<0.001	<0.001	<0.001	0.27
Ocular pain	86.4 (0–100), 2	89.2 (25–100), 1	89.5 (38–100), 2	0.2	0.26	0.25	0.9
Near activities	63.4 (8–100), 2	92.2 (50–100), 1	93.7 (46–100), 2	<0.001	<0.001	<0.001	0.6
Distance activities	61.55 (4–100), 2	93.78 (46–100), 1	95.5 (58–100), 2	<0.001	<0.001	<0.001	0.6
Social functioning	81.2 (8–100), 2	98 (67–100), 1	99.17 (75–100), 2	<0.001	<0.001	<0.001	0.62
Mental health	66.9 (0–100), 3	90.9 (10–100), 1	91.9 (60–100), 2	<0.001	<0.001	<0.001	0.76
Role difficulties	69.1 (0–100), 2	94.1 (25–100), 1	95.5 (50–100), 2	<0.001	<0.001	<0.001	0.73
Dependency	83.6 (0–100), 3	96.9 (0–100), 1	99.6 (88–100), 2	<0.001	<0.001	<0.001	0.37
Driving	37.6 (0–100), 2	81.2 (0–100), 1	75.2 (0–100), 3	<0.001	<0.001	<0.001	0.28
Colour vision	87 (0–100), 2	99.1 (75–100), 1	99.2 (75–100), 2	<0.001	<0.001	<0.001	0.95
Peripheral vision	78.2 (0–100), 2	95.5 (25–100), 1	96.9 (50–100), 2	<0.001	<0.001	<0.001	0.63

In children with *OPA1* mutation, visual ability expressed as the median CVAQC logit score was significantly lower than the score in first‐degree relatives (p = 0.037, Table 4).

**Table 4:** Cardiff Visual Ability Questionnaire for Children scores.

	Patients	First‐degree relatives	p
CVAQC score (median)	−1.4	−2.7	0.037
Interquartile range	−1.9 to −0.6	−2.8 to −2.5	

**Fig. 2:**
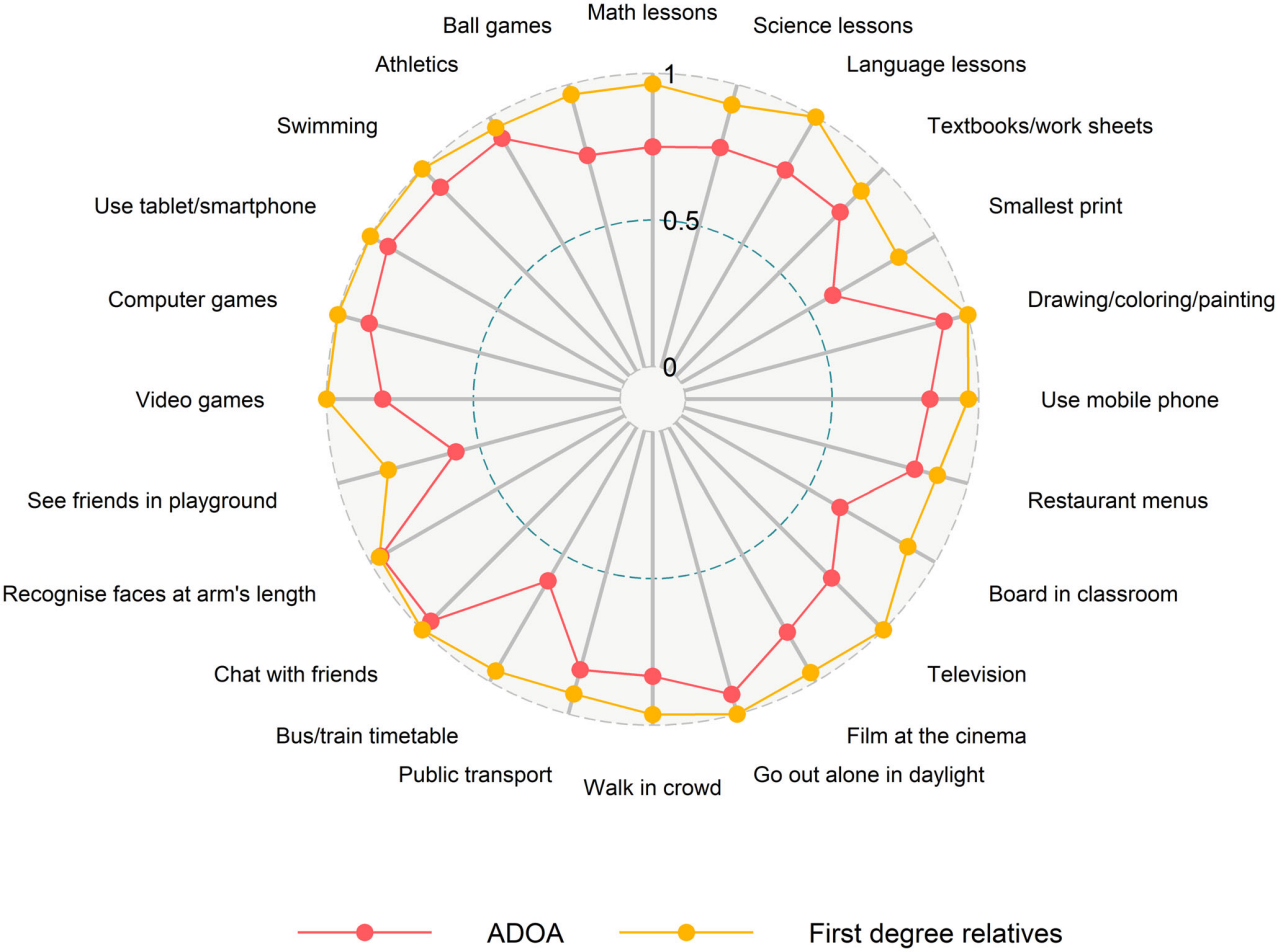
Cardiff Visual Ability questionnaire for Children (CVAQC) ratio scores on visual ability in children with *OPA1* mutation (red) and in healthy first‐degree relatives (yellow) on arbitrary scales from 0 (worst) to 1 (best). [Colour figure can be viewed at wileyonlinelibrary.com]

Subscale items with the most prominent deficits in children with *OPA1* mutation were the educational activities *science lessons* (p = 0.046) and *language lessons* (p = 0.038) and *reading the smallest text in a textbook*, *watching television* and *reading a bus or train timetable at a station* were trending in the same direction (Table 5, Fig. 2).

**Table 5:** Mean and range ratios of the CVAQC questionnaire subscales. [Correction added on 25 March 2022, after first online publication: The mean values for first‐degree relatives in Table 5 were corrected in this version.] [Correction added on 12 April 2022, after first online publication: Parentheses were added for all mean values in this version.]

	ADOA (*n* = 18)		First‐degree relatives (*n* = 7)		p
	Mean, (n/a)	Range	Mean, (n/a)	Range	
Math lessons	0.75, (0)	0.25–1	0.96, (0)	0.75–1	0.12
Science lessons	0.78, (0)	0.5–1	0.93, (0)	0.5–1	0.046
Language lessons	0.79, (0)	0.5–1	1, (0)	1–1	0.038
Reading textbooks and work sheets in school	0.79, (0)	0.5–1	0.9, (0)	0.5–1	0.2
Reading the smallest print in textbook	0.58, (0)	0.25–1	0.9, (0)	0.5–1	0.052
Drawing, colouring or painting	0.92, (0)	0.5–1	1, (0)	1–1	0.3
Reading text messages on mobile phone	0.83, (0)	0.25–1	0.96, (0)	0.75–1	0.09
Reading restaurant menus	0.81, (2)	0.5–1	0.89, (0)	0.5–1	0.2
Reading the board in classroom	0.63, (0)	0.25–1	0.89, (0)	0.5–1	0.28
Watching television	0.75, (0)	0.5–1	1, (0)	1–1	0.052
Watching film at the cinema	0.81, (0)	0.5–1	0.96, (0)	0.75–1	0.22
Going out alone in daylight	0.93, (0)	0.75–1	1, (0)	1–1	0.28
Walking in crowd	0.82, 0)	0.25–1	0.96, (0)	0.75–1	0.13
Using public transport (bus/train)	0.84, (2)	0.5–1	0.93, (0)	0.75–1	0.32
Reading a bus/train timetable at a station	0.6, (1)	0.25–1	0.96, (1)	0.75–1	0.055
Chatting with friends	0.96, (0)	0.75–1	1, (0)	1–1	0.47
Recognizing faces at arm's length	0.96, (0)	0.5–1	0.96, (0)	0.75–1	0.5
Seeing a friend in a playground	0.58, (0)	0.25–1	0.82, (0)	0.5–1	0.16
Playing video games	0.82, (1)	0.5–1	1, (1)	1–1	0.18
Playing computer games	0.88, (0)	0.5–1	1, (0)	1–1	0.2
Using a tablet or smartphone	0.92, (0)	0.5–1	1, (0)	1–1	0.28
Swimming	0.91, (1)	0.5–1	1, (1)	1–1	0.3
Taking part in athletics	0.92, (3)	0.75–1	0.96, (1)	0.75–1	0.6
Playing ball games	0.75, (0)	0.25–1	0.96, (0)	0.75–1	0.1

Among adults with *OPA1* mutation, both the composite NEI‐VFQ‐39 score and all subscale scores decreased with decreasing visual acuity and with poorer contrast sensitivity. Location of the fixation was significantly associated with both composite score and all subscale scores except general health, ocular pain, mental health, dependency, colour vision and peripheral vision. Fixation stability and area of preferred fixation was significantly associated with both composite score and all subscale scores except general health (Table 6).

**Table 6:** Correlation of NEI‐VFQ‐39 scores with visual acuity and contrast sensitivity in adults with *OPA1* mutation.

	Visual acuity (ETDRS letters)		Contrast sensitivity (log CS)		Fixation location (reference: central)		Fixation stability (reference: stable)		Fixation area (°^2^)	
	Estimate (95% CI)	p	Estimate (95% CI)	p	Estimate (95% CI)	p	Estimate (95% CI)	p	Estimate (95% CI)	p
Composite score	0.7 (0.6;0.8)	<0.001	46.3 (35.3;57.3)	<0.001	−16.3 (−24.8;−7.8)	<0.001	−20.9 (−27.9;−13.8)	<0.001	−0.9 (−1.3;−0.6)	<0.001
General health	0.2 (N/A)	0.02	19.4 (6.1;32.8)	0.005	−2.95 (−11.8;6)	0.5	−3,2 (−11.1;4.7)	0.4	−0.31 (−0.7;0.05)	0.0905
General vision	0.8 (0.7;1)	<0.001	54.7 (41;68.3)	<0.001	−20.5 (−30.5;10.5)	<0.001	−26.3 (−34.5;−18)	<0.001	−1 (−1.4;−0.6)	<0.001
Ocular pain	0.3 (N/A)	<0.001	19.6 (6.8;32.3)	0.003	−7.2 (−16;1.6)	0.1	−10.1 (N/A)	0.0106	−0.5 (N/A)	0.0106
Near activities	0.9 (0.7;1)	<0.001	54.6 (40.2;69)	<0.001	−28.9 (N/A)	<0.001	−30.7 (N/A)	<0.001	−1.2 (N/A)	<0.001
Distance activities	0.9 (0.8;1.1)	<0.001	60.5 (47;74)	<0.001	−23 (N/A)	<0.001	−26.5 (−35;−18)	<0.001	−1.1 (−1.5;−0.6)	<0.001
Social functioning	0.7 (N/A)	<0.001	40.4 (N/A)	<0.001	−17.4 (−25.8;−8.9)	<0.001	−20.3 (N/A)	<0.001	−0.8 (N/A)	<0.001
Mental health	0.6 (N/A)	<0.001	37.4 (21.4;53.5)	<0.001	−5.4 (−16.7;5.9)	0.3	−12.5 (−22.4;−2.6)	0.0138	−0.7 (−1.2;−0.3)	0.0031
Role difficulties	0.8 (0.6;1)	<0.001	52 (36.9;67)	<0.001	−17 (−27.8;−6)	0.0028	−22.3 (−31.6;−13)	<0.001	−1.1 (−1.5;−0.6)	<0.001
Dependency	0.4 (N/A)	<0.001	31.2 (18.2;44.3)	<0.001	−7.8 (−17.1;1.5)	0.0983	−13.5 (N/A)	0.0012	−0.7 (N/A)	<0.001
Driving	1.5 (1.2;1.7)	<0.001	92.8(68.7;116.9)	<0.001	−36.1 (−53.2;−19.1)	<0.001	−41.4 (−56;−26.9)	<0.001	−1.6 (−2.4;−0.9)	<0.001
Colour vision	0.4 (0.3;0.6)	<0.001	26.9 (14;39.8)	<0.001	−5.4 (−14;7.4)	0.2	−11.7 (−19.1;−4.2)	0.0026	−0.6 (−0.9;−0.2)	0.0010
Peripheral vision	0.6 (0.4;0.7)	<0.001	36.9 (22.3;51.6)	<0.001	−9 (−19.4;1.5)	0.0906	−13.9 (−23;−4.8)	0.0033	−0.7 (−1.2;−0.3)	0.0012

**Fig. 3:**
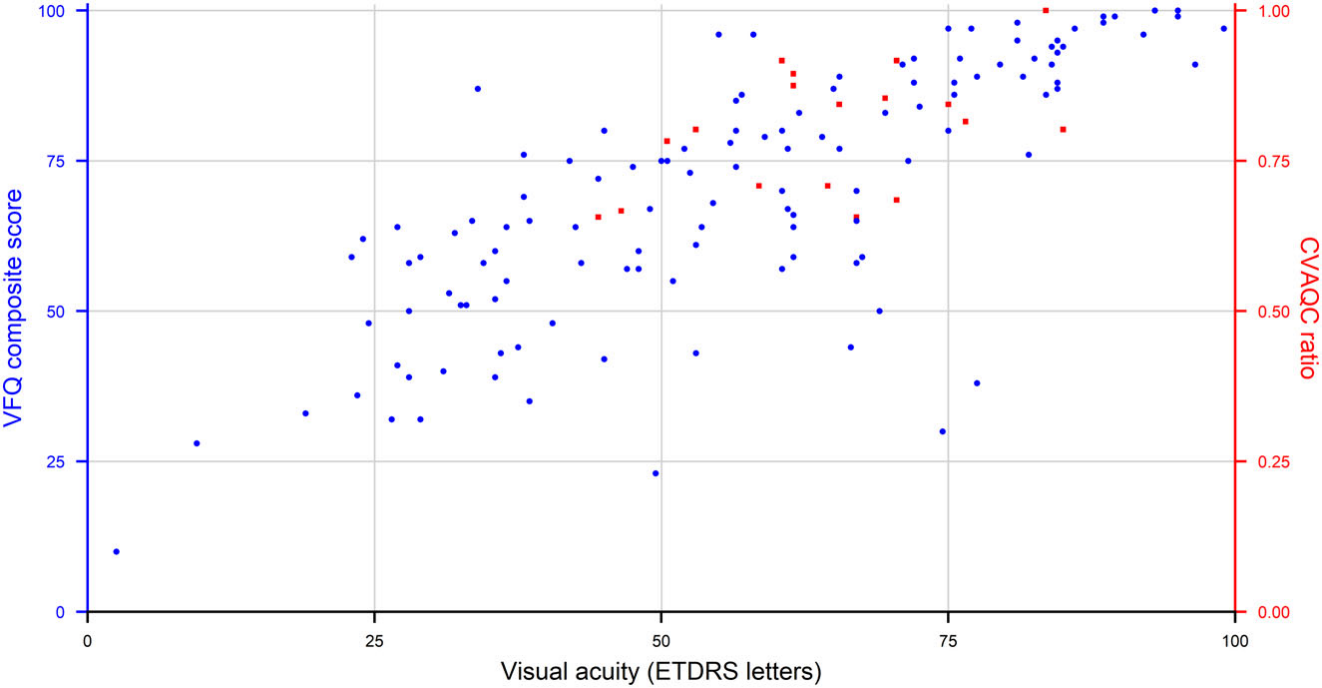
Association between VFQ‐39 composite score in adults (blue) and CVAQC ratio in children (red) and average visual acuity in ETDRS letters. [Colour figure can be viewed at wileyonlinelibrary.com]

**Fig. 4:**
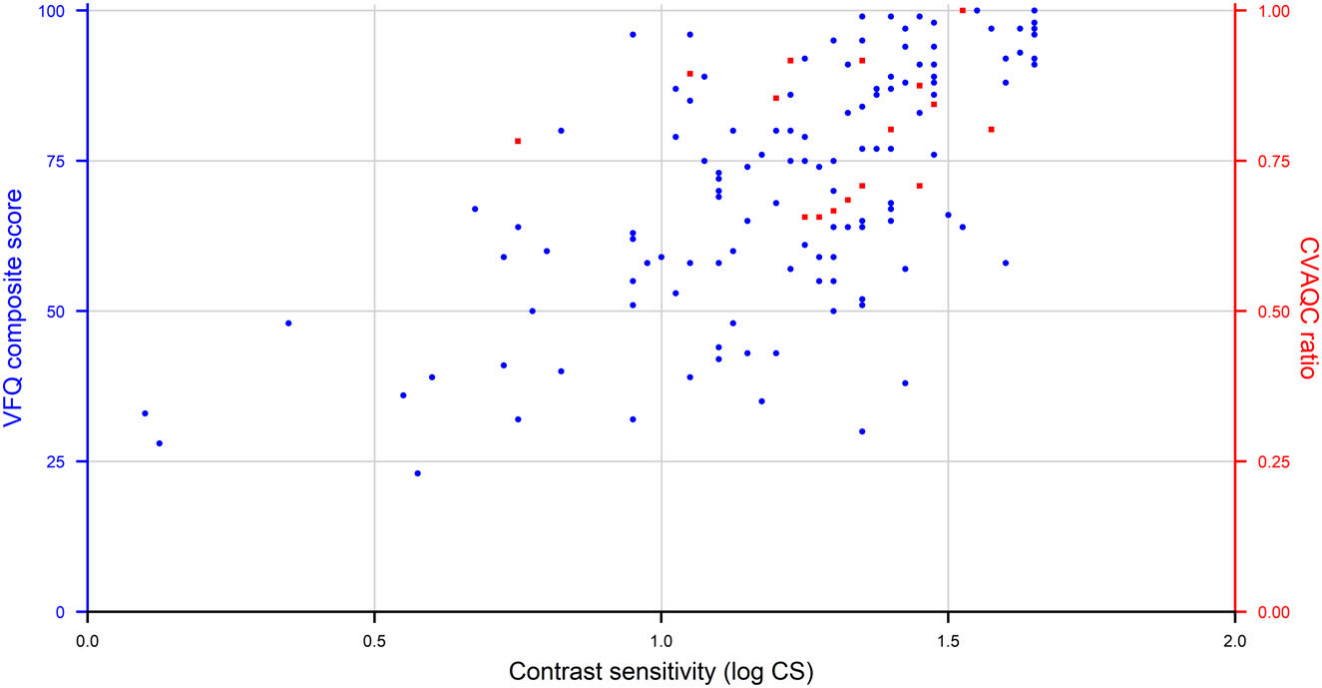
Association between VFQ‐39 composite score in adults (blue) and CVAQC ratio in children (red) and average contrast sensitivity in log CS. [Colour figure can be viewed at wileyonlinelibrary.com]

The relatively small number of children in the study did not permit a meaningful analysis of statistical significance between CVAQC scores and visual acuity or contrast sensitivity, but similar trends as in adults are discernible (see Fig. 3 for visual acuity and Fig. 4 for contrast sensitivity).

## Discussion

This study of vision‐related quality of life and visual ability in adults and children with pathogenic *OPA1* mutations identified specific activities of daily life that are impaired by having ADOA and showed that the severity of impairment is closely related to the reduction in both best‐corrected visual acuity, contrast sensitivity and location and stability of fixation.

Study participants with ADOA in this study had reduced quality of life from difficulties with a broad range of activities of daily life, but for adults, reduction or loss of the ability to drive a car was the most prominent problem. This information supports that maintenance of driving vision is valuable benefit of treatment ‐ should a treatment for ADOA become available, and one that is more easily attainable than perfect vision. For children, the study found that educational activities involving small print or complex graphics and equations are particular challenges. This information can be used to focus didactic efforts and the use of compensatory remedies.

Decreased quality of life has been demonstrated in various hereditary eye diseases. Sahli *et al*. (2020) found an overall score of the NEI‐VFQ‐25 questionnaire of 54.3 in patients with cone dystrophy and an overall score of 44 for patients with Stargardt disease, which is lower than the overall composite score of 69.7 in our ADOA study population. In patients with retinitis pigmentosa, Azoulay *et al*. reported a median composite NEI‐VFQ‐25 score of 37.7 in patients with a Snellen VA ≤ 0.3, compared with 55.9 in patients with Snellen VA of 0.3 to 0.7 and 60.8 in patients with Snellen VA > 0.7. In the control subjects of the study, the median composite NEI‐VFQ‐25 score was 93.7, which is comparable to the control subjects in the present study (Azoulay *et al*. 2015). Sugawara *et al*. (2011) found a mean composite NEI‐VFQ‐25 score of 69.4 for patients with retinitis pigmentosa. While these findings suggest that ADOA patients may have a better overall composite score and vision‐related quality of life compared to patients with other causes of subnormal visual acuity, recruitment bias may explain some of the differences between the study populations. Thus, our recruitment was family‐based and hence enabled inclusion of asymptomatic family members with normal visual acuity. Nevertheless, there may also be effect of the density of the central scotoma, the involvement of receptive fields, the time of onset in relation to visual maturation and the manner of compensation (*e.g*. the choice of eccentric locus of fixation). Thus, many of the participants in the present study had central fixation, whereas the patients with cone dystrophy and Stargardt disease studied by Sahli *et al*. had an eccentric fixation. The understanding of differences in vision‐related quality of life between people with different eye diseases is obviously limited by the methods available for the mapping of visual function in daily life.

Hahm *et al*. (2008) found a decrease in all subgroup scores in patients with retinitis pigmentosa compared to the patients with ADOA in this study. In Stargardt disease, Murro *et al*. (2017) showed decreased subscale scores from 45 to 56 for all items except social functioning (71) and colour vision (100). In glaucoma, Orta *et al*. (2015) found mean scores to be decreased in most subscales of between 55 and 82, except from social functioning (91) and colour vision (92). On most subscales, patients with ADOA are comparable to the patients with retinitis pigmentosa, Stargardt disease and glaucoma in the studies by Sugawara *et al*., Murro *et al*. and Orta *et al*.. On subscales, ADOA patients scored better than Stargardt patients and worse than patients with glaucoma. The only subscale where ADOA had the best score was dependency, again suggesting that their psychophysical and visual coping mechanisms produce a relatively favourable outcome.

When comparing the median logit score of the child group in the present study to other patient groups, patients with ADOA have a vision‐related quality of life (−1.4, IQR −1.9 to −0.6) that is similar to other patient groups including children with glaucoma with a reported median score of −1.24 (IQR −2.2 to −0.11) (Dahlmann‐Noor *et al*. 2017) and −0.68 (IQR −1.27 to 0.19) (AlDarrab *et al*. 2019), children with microphthalmia/anophthalmia/coloboma (MAC) with a reported median score of −1.4 (IQR −2.4 to 0.4) (Dahlmann‐Noor *et al*. 2018) and children treated for cataract with a reported median score of −1.42 (IQR −2.28 to −0.03) (Tailor *et al*. 2017).

As our clinical experience is that patients with ADOA manage well in life despite their decreased visual function, one of the reasons are thought to be early diagnosis, in most cases when the patients begin elementary school. This might result in an increased awareness of the patients' disabilities, that leads to a development of alternative skills in everyday management. The study showed a decrease in vision‐related quality of life similar to other patient groups with various visual disabilities, which suggests that patients with ADOA do not necessarily have a better quality of life despite early onset of visual disability, but might feel more independent regarding help from family members, friends, colleagues *etc*. In adults, first‐degree relatives scored higher than unrelated controls on the subscales of general vision and driving, which might be caused by patients and first‐degree relatives growing up in the same environment, leading to a broader consciousness of the visual problems their relatives are experiencing.

A strength of the study is inclusion of a large patient group, of whom 145 out of 158 (91%) were genetically verified. Of the included adults and children, 104 out of 145 (72%) had one of the 3 major mutations found in the study. Another strength is a single examiner having executed all examinations, which reduces measurement bias. Limitations of the study is the age of the ADOA patients not being comparable to the control groups and having different versions of instruments being used at the two examination sites. Another limitation is that no perimetry additional to the microperimetry was performed, for assessing the type of scotoma. Although adult participants with *OPA1* mutation were older than the mutation‐free first‐degree relatives and the unrelated control subjects, the age‐distribution was wide enough for all three groups to provide a solid base for age‐corrected analyses. The number of participants in the group of children was limited, with 18 affected children and 7 unaffected first‐degree relatives; thus, the results should be interpreted with caution. The relatively small number of children included in the study did not permit a meaningful analysis of statistical significance between CVAQC scores and visual acuity or contrast sensitivity. Despite the low number of participants, a mixed model was necessary, as family relations needed to be taken into account. An additional limitation of the study is the questionnaires not being validated for the particular use in study participants with ADOA, as the Danish version of the NEI‐VFQ‐39 is validated in patients with age‐related macular degeneration. There might be limitations associated with the method of summing up the individual, ordinal scores and treating them as continuous variables. A few participants with verified *OPA1* mutations had normal vision and normal contrast sensitivity and hence did not have the ADOA phenotype. Vision‐related quality of life in the study population was strongly correlated with VA and other visual function parameters (see Table 6 and Figs. 3 and 4). Thus, it is the very wide variation in visual performance in ADOA, ranging from 3 to 99 ETDRS letters in our study population and not the ageing within the span of 7–86 years, that is the major factor behind the recorded vision‐related quality of life deficit. In adults, ADOA is associated with an overall decrease in vision‐related quality of life that is correlated with both visual acuity, contrast sensitivity and location and stability of fixation, which is comparable to other hereditary ocular pathologies.

In conclusion, both visual function and quality of life is decreased in both adults and children with ADOA but is comparable to patients with hereditary ophthalmic diseases. Mutations in the *OPA1* gene might cause a lower visual function, which is associated with a lower quality of life and visual ability in patients with autosomal dominant optic atrophy.
